# Bioprospecting of *Helichrysum* Species: Chemical Profile, Phytochemical Properties, and Antifungal Efficacy against *Botrytis cinerea*

**DOI:** 10.3390/plants12010058

**Published:** 2022-12-22

**Authors:** Neliswa A Matrose, Zinash A Belay, Kenechukwu Obikeze, Lucky Mokwena, Oluwafemi James Caleb

**Affiliations:** 1 Post-Harvest and Agro-Processing Technologies (PHATs), Agricultural Research Council (ARC) Infruitec-Nietvoorbij, Stellenbosch 7599, South Africa; 2 School of Pharmacy, Faculty of Science, University of the Western Cape, Bellville 7535, South Africa; 3 Central Analytical Facility, Stellenbosch University, Matieland 7602, South Africa; 4 Department of Food Science, Faculty of AgriSciences, Stellenbosch University, Matieland 7602, South Africa; 5 African Institute for Postharvest Technology, Faculty of AgriSciences, Stellenbosch University, Private Bag X1, Matieland 7602, South Africa

**Keywords:** antioxidant capacity, crude extracts, extraction solvents, ‘imphepho’, secondary metabolites, sesquiterpenoids

## Abstract

Variation in plant species and extraction solvents play a crucial role in the recovery of their bioactive compounds and antifungal efficacy. Thus, in this study, a comparative investigation was carried out using extraction solvents: 70% acetone and 95% ethanol to obtain crude aqueous extracts from *Helichrysum odoratissimum* and *H. patulum*. Crude aqueous extracts were screened using gas chromatography–mass spectrometry (GC–MS), to gain insight into their chemical composition. Phytochemical properties (total polyphenols (TP) and radical scavenging capacity via 2,2-diphenyl-1-picrylhydrazyl (DPPH)), and antifungal activity against *Botrytis cinerea* of the crude extracts were evaluated. Fungicide (Rovral^®^ WP) and extraction solvents were used as controls. Variation in *Helichrysum* spp. and extraction solvent had influence on the chemical composition, phytochemicals, and antifungal activities. Metabolites such as γ-terpinene (≈0.1%), α-amorphene (≈0.6%) α-gurjunene (≈1.4%), β-selinene (2.2–3.2%), γ-gurjunene (≈3.3%), and methyl cinnamate (≈20%) were detected only in extracts of *H. patulum*. Crude extract of *H. odoratissimum* using 70% acetone had the highest TP (19.3 ± 0.76 g GA 100 g^−1^), and DPPH capacity (13,251.5 ± 700.55 µmol Trolox g^−1^) compared to *H. patulum* (*p* ≤ 0.05). Ethanolic extracts of *H. patulum* showed highest antifungal efficacy (≈65%) against *B. cinerea* (*p* ≤ 0.05) compared to other crude extracts. This study showed that *Helichrysum* spp. differ in their potential as a source for bioactive compounds and antifungal treatments/formulations.

## 1. Introduction

Crude extracts of medicinal plants have been demonstrated as rich sources of bioactive molecules with curative capability. This curative potential is attributed to the complex mixtures of these bioactive compounds of different chemical classes, with antioxidant and antimicrobial activity [1]. Allopathic medicine is emerging as a new field of research globally as potential source of novel active/functional compounds for new drugs [2]. In the agricultural sector, disease-causing fungal pathogens result in serious deterioration of fresh horticultural commodities after leaving the farm gate and during storage. Synthetic fungicides and bio-based fungicides have been successful in mitigating the impact of these pathogens [1,3]. According to the Fungicides Global Market Report [4], it is estimated that the global fungicides market will grow from 2021 value of approximately $19.32 billion to $20.64 billion in 2022, and to $26.09 billion in 2026. This is a huge cost for the management of agricultural produce. Therefore, bioprospecting for new natural bioactive compounds with anti-fungal properties as alternatives to existing fungicides is crucial for the development biocontrol treatment of fungal pathogens.

*Helichrysum* (Asteraceae), locally known in South African traditional/folk medicine by a collective name as ‘*Imphepho*’, is one of the mostly used species to combat ailments, such as the treatment of wounds, burns, pimples, eczema, as well as coughs and colds [5,6]. Traditional uses of the species also include the treatment of abdominal pains, catarrh, headache, fever, menstrual disorders, and urinary tract infections, suggesting that these plants parade anti-microbial and -inflammatory effects [7]. About 245–250 species, from the genus consisting of roughly 500–600 species, are indigenous to South Africa [1,7].

Several studies have reported on different pharmacological properties of the essential oils (EOs) extracted from *Helichrysum* spp. Adewingo et al. [8] explored the biological activities of EO extracted from *H. petiolare*, *H. odoratissimum*, and *H. cymosum* and found out that the EOs possessed low antibacterial, anti-tyrosinase activity but promising antioxidant capacity. The study conducted by Najar et al. [8] demonstrated a good antimicrobial activity for EOs of *H. pandurifolium* and *H. trilineatum* whereas, EOs from *H. edwardsii* and *H. pandurifolium* did not show any antimicrobial activity. A recent study by Serabele et al. [9] investigated the chemical profiling and antimicrobial activity of aqueous methanol of *H. odoratissimum* and *H. petiolare*. The authors reported significantly higher antimicrobial effects of the methanolic extracts obtained from *H. odorutissimum* towards a larger range of bacteria in comparison to *H. petiolare* extracts with minimum inhibitory concentration (MIC) of 0.13 mg mL^−1^ < MIC ≤ 1000 µg mL^−1^ and 4.0 mg mL^−1^ < MIC ≤ 1000 µg mL^−1^, respectively. The highest antimicrobial effect of the methanolic extracts was against *Escherichia coli*. Furthermore, based on ultra-performance liquid chromatography–mass spectrometry (UPLC–MS) analysis, the authors also observed high chemical variation within the two species.

Moreover, Zantanta et al. [10] studied the activities of *H. odoratissimum* plants cultivated via aquaponics and hydroponics and found out that *H. odoratissimum* plants cultivated via aquaponics exhibited the bet antifungal activity whereas those cultivated via hydroponics yielded the highest antioxidant activity. A gas chromatography–mass spectrometry analysis performed by Acimovic et al. [11] on EO of *H. italicum* detected a total number of 70 EO constituents. This was in support of a previous study by Djihane et al. [12] who investigated the chemical constituents of *H. italicum* EO obtained through hydro-distillation of air-dried and crushed aerial parts of the plant and their antimicrobial activity against gram-positive and gram-negative bacteria, filamentous fungi, and *Candida albicans*. The study reported a 0.44% (*v*/*w*) EOs yield and the presence of 67 compounds (accounting for 99.24% of the oil) with oxygenated sesquiterpenes making up 61.42% of the compounds. Furthermore, a study by Lawal et al. [13] reported that monoterpene hydrocarbons (72.9%) and sesquiterpene hydrocarbons (15.6%) were the prominent class of compounds present in the EOs (obtained through hydro-distillation of air-dried and crushed leaves) of the Spp. The authors suggested that *Helichrysum* spp. could be classified based on monoterpene hydrocarbons and sesquiterpene hydrocarbons.

*Helichrysum* spp. has been reported as an ethnopharmacological treasure of bioactive compounds. Matrose et al. [1] investigated the impact of spatial variation in *H. odoratissimum* and different extraction solvents (95% ethanol and 70% acetone) on extractable yield (soluble solids), total polyphenol content (TP), antioxidant (via 2,2-diphenyl-1-picrylhydrazyl (DPPH)), and antifungal activity. Their study showed that acetone extracts had the highest soluble solids (SS), TP, and DPPH capacity (*p* < 0.05) in comparison with that of ethanol. However, the authors did not account for the impact of species variation in the performance of crude extract. Bioprospecting the *Helichrysum* spp. is crucial to the construction of a high-quality database for their crude extract, structural diversity of their secondary metabolites, active compounds, and antifungal/antimicrobial activities. Thus, the set hypothesis for this study was that (*i*) extraction solvents cannot influence the extractable yield of the *Helichrysum* spp., and (*ii*) the antioxidant and antimicrobial activities of *Helichrysum* spp. would be influenced by the chemical composition. Therefore, the aim of this study was the comparative analysis of acetone and ethanolic extracts of *H. odoratissimum* and *H. patulum*. The specific objectives were to compare the secondary metabolite profile and phytochemical properties, as well as the antifungal efficacy of these crude extracts obtained from *H. odoratissimum* and *H. patulum*.

## 2. Results

### 2.1. Soluble Solids

The results of this study showed that only extraction solvents had significant (*p* < 0.000) effects on the soluble solid (SS) content of both *Helichrysum* species (Table 1). For example, soluble solid yield for *H. odoratissimum* obtained from the acetone extract (2.9 ± 0.003 g 100 mL^−1^) was significantly higher than ethanol (1.6 ± 0.002 g 100 mL^−1^). A similar pattern was observed for *H. patulum*, where the SS of 2.9 ± 0.003 g 100 mL^−1^ and 1.6 ± 0.002 g 100 mL^−1^ was found for acetone and ethanol extracts, respectively (Table 1).

### 2.2. Total Polyphenol Content (TP)

Table 1 presents the TP content of acetone and ethanol extracts of *H. odoratissimum* and *H. patulum*. The interactive of species and extraction solvent had a significant impact of the TP content, with highest TP content (19.3 ± 0.76 g GA 100 g^−1^) found in acetone extracts of *H. odoratissimum* (*p* < 0.000, DF = 8, F = 2144.5). Overall, TP content for acetone and ethanolic extracts of *H. odoratissimum* (19.3 ± 0.76 g GA 100 g^−1^ and 14.5 ± 0.45 g GA 100 g^−1^, respectively) were significantly higher than that of *H. patulum* for both extracts (4.9 g GA 100 g^−1^ ± 0.22 and 4.4 ± 0.09 g GA 100 g^−1^), respectively. These findings were consistent with literature, as previous studies have also reported that different extraction solvents significantly affected the extraction yield of TP [7,9].

### 2.3. Antioxidant Activity

The results obtained in this study showed that, the DPPH scavenging capacity *of H. odoratissimum* and *H. patulum* extracts were strongly dependent on the extraction solvents (Table 1). Generally, *H. odoratissimum* had highest free radical scavenging capacity (13,252 ± 701 μmol TE g^−1^ and 9281 ± 704 μmol TE g^−1^) for the extracts obtained with 70% acetone and 95% ethanol, respectively (*p* ≤ 0.05). In contrast, extracts from *H. patulum* showed lower scavenging activity (5643 ± 259 μmol TE g^−1^ and 2926 ± 139 μmol TE g^−1^, for 70% acetone and 95% ethanol, respectively) at *p* ≤ 0.05. It is also noteworthy that acetone was the better solvent for antioxidant activity for both *H. odoratissimum* and *H. patulum*.

### 2.4. Volatile Compounds Analysis

Gas chromatography–mass spectrometry (GC–MS) results of the volatile compounds obtained from *Helichrysum* crude extracts are shown in Table 2. In this study, a total of 71 volatile compounds were tentatively identified across both extraction solvents. These compounds were classified into 16 chemical classes. Based on the result obtained, the contribution of individual chemical classes from the 70% acetone and 95% ethanol extracts of *H. odoratissimum* differed quantitatively (*p* ≤ 0.05). Acetone extract of *H. odoratissimum* contained the most abundant volatiles with sesquiterpenoids (40.2%) > alkanes (10.6%) > aromatic hydrocarbons (10.2%) > sesquiterpenes (8.7%) > monoterpenoids (4.3%) > monoterpenes (1.8%) > terpene (1.2%) > epoxide (0.8%) > phenylpropene and terpenoid (0.3%) > bromodiphenyl ethers (0.2%). On the other hand, the most abundant classes volatile compounds in ethanolic extracts of *H. odoratissimum* were in the following order: sesquiterpenoids (37.8%) > sesquiterpenes (18.6%) > oxanes (14%) > alkane hydrocarbons (12.4%) > terpene (3.3%) > monoterpenes (1.9%) > terpenoid (1.6%) > epoxide, phenylpropene, and bromodiphenyl ethers (0.7%) > monoterpenoids (0.6%) > aliphatic compounds (0.3%). A similar pattern was obtained for *H. patulum* for each given solvent with lesser quantities of mostly commonly identified compounds.

Sesquiterpenoids are modified class of terpenes with different functional groups and oxidized methyl groups moved or removed at various positions with three carbon isoprene units. These terpene derivatives are equipped with high pharmacological properties [14]. Thus, their presence in plant extracts signals its potential therapeutic effects. Viridiflorol, a sesquiterpenoid, was the major compound across all *Helichrysum* spp. and both extraction solvent tested. This study showed the relative percentage/concentration of viridiflorol (26.2 ± 1.24% and 16.8 ± 0.31%) for acetone and ethanol extracts of *H. odoratissimum*; the relative percentage/concentration of viridiflorol (17.9 ± 2.15%) for acetone and (17.0 ± 0.01%) ethanol extracts of *H. patulum* extracts were significantly (*p* < 0.05) lower than the acetone extracts of *H. odoratissimum*. Relatively, higher concentrations of alkane hydrocarbons such as tetradecane and hexadecane were identified for *H. odoratissimum* extracts regardless of extraction solvents compared to *H. patulum.*

Furthermore, compounds such as xylene, para-xylene, and α-pinene oxide were only identified under acetone extracts of *H. odoratissimum*, whereas a monoterpene: 1,8-Cineole (percentage: 13.6 ± 0.52%) was only recorded in the ethanol extract of *H. odoratissimum*. Relative percentage of sesquiterpenes: α-gurjunene (1.2 ± 0.19% and 1.4 ± 0.08%), β-caryophyllene (1.2 ± 0.16% and 1.3 ± 0.03%), β-selinene (2.2 ± 0.20% and 3.2 ± 0.01%), and γ-gurjunene (3.2 ± 0.57% and 3.3 ± 0.08%), were only identified in *H. patulum* for both acetone and ethanol solvents, respectively. Similarly, a significantly higher (*p* < 0.05) concentration of a fatty acid methyl ester methyl cinnamate (cinnamic acid, methyl ester), which is a sesquiterpene, was only identified in the acetone and ethanol extracts of *H. patulum* (20.6 ± 0.03% and 19.7 ± 0.05%, respectively). On the contrary, δ-cadinene was identified as the second major compounds under the sesquiterpenoid category, however, significantly high concentrations of δ-cadinene (20.4 ± 1.30% and 16.1 ± 0.49%) were quantified in the acetone and ethanol extracts of *H. patulum* extracts than those of *H. odoratissimum* (5.5 ± 0.46% and 2.1 ± 0.20%).

**Table 2:** Relative percentage composition of volatile compounds isolated from crude acetone and ethanol extracts of *H. odoratissimum* (H. od) and *H. patulum* (H. pat) from Western Cape, South Africa.

Classification	Compounds	RT	RI (Exp.)	RI (Lit.) ^†^	Samples and Extraction Solvent			
					Acetone		Ethanol	
					*H. od* (%)	*H. pat* (%)	*H. od* (%)	*H. pat* (%)
* AH	Undecane	16.39	180.44	-	1.1 ± 0.36 A	0.4 ± 0.16 C	1.8 ± 0.35 A	0.5 ± 0.05 B
	Dodecane (CAS)	21.83	200.12	-	4.3 ± 0.29 A	0.8 ± 0.17 C	3.4 ± 0.39 B	0.9 ± 0.06 C
	Tetradecane (CAS)	33.14	236.21	-	3.2 ± 0.69 B	1.1 ± 0.20 C	4.1 ± 0.06 A	nd
	Hexadecane (CAS)	43.08	268.29	-	0.7 ± 0.02 B	nd	1.8 ± 0.05 A	nd
	4,5-dimethyl-11-methylenetricyclo [7.2.1.0 (4.9)] dodecane	45.42	n/a	-	0.1 ± 0.00 B	nd	0.8 ± 0.09 A	nd
*Alkylbenzene*	Benzene, 1,2,3,5-tetramethyl- (CAS)	35.18	1129	1116 D	0.9 ± 0.00 A	0.1 ± 0.02 B	nd	nd
	Benzene, 1,2,4,5-tetramethyl- (CAS)	34.68	1125	1093 D	1.1 ± 0.02	nd	nd	nd
	Benzene, 1,2,4-trimethyl-	27.32	1006	1004 D	0.2 ± 0.08	nd	nd	nd
*Aromatic ether*	Diphenyl ether	53.12	1525.0	1396 C	0.2 ± 0.00 B	0.1 ± 0.11 B	0.6 ± 0.14 A	nd
* Aroma. Hyds	Toluene	13.64	785.29	-	0.4 ± 0.04 A	0.1 ± 0.02 B	nd	nd
	Benzene, ethyl- (CAS)	18.51	850.6	868 C	0.4 ± 0.08 A	0.1 ± 0.03 B	nd	nd
	Para-xylene	19.02	852.1	-	0.8 ± 0.01	nd	nd	nd
	M-xylene	19.42	848	-	3.8 ± 0.01 A	1.1 ± 0.18 B	nd	nd
	O-Xylene	22.05	909	881 D	1.4 ± 0.69	nd	nd	nd
* BH	6,7-Dimethyltetralin-1,5,8-trione	44.72	n/a	-	nd	nd	nd	4.5 ± 0.18
*Epoxide*	α-Pinene oxide	48.63	949	1084 C	0.3 ± 0.08	nd	nd	nd
	Caryophyllene oxide	52.43	1578	1578 A	0.2 ± 0.01 B	0.2 ± 0.03 B	0.5 ± 0.16 A	0.7 ± 0.13 A
* FAME	* Methyl cinnamate	52.00	1399	1379 C	nd	20.6 ± 0.03 A	nd	19.7 ± 0.05 B
*Monoterpenes*	β-pinene	16.90	986	981 A	0.8 ± 0.08 B	1.2 ± 0.22 AB	0.9 ± 0.18 B	1.3 ± 0.10 A
	Limonene	22.31	1025	1029 B	0.8 ± 0.01 A	0.3 ± 0.05 B	0.7 ± 0.07 A	0.3 ± 0.02 B
	1,8-Cineole (Eucalyptol)	22.96	1028	1029 B	nd	nd	13.6 ± 0.52	nd
	γ-terpinene	25.00	1056	1063 A	nd	0.1 ± 0.03 A	nd	0.1 ± 0.06 A
	Trans-β-ocimene	24.60	1040	1037 B	nd	0.2 ± 0.05	nd	nd
	Cis-β-ocimene	24.70	1095	1057 A	nd	nd	nd	0.4 ± 0.05
	o-cymene	26.54	1029	1032 C	1.8 ± 0.35 A	0.1 ± 0.02 C	0.5 ± 0.03 B	nd
	Para-cymene	26.76	1018	1015 C	0.5 ± 0.02 A	nd	nd	0.2 ± 0.01 B
	Neo-allo-ocimene	45.19	1135	1130 C	0.2 ± 0.00 A	nd	0.2 ± 0.12 A	nd
*Monoterpenoids*	α-terpinolene	27.10	1083	1079 C	0.4 ± 0.07 A	0.1 ± 0.01 B	nd	nd
	Verbenyl ethyl ether	31.90	1382	-	nd	nd	nd	0.3 ± 0.01
*Naphthalenes*	Naphthalene,1,2-dihydro-1,1,6-trimethyl	52.40	1454	-	nd	0.2 ± 0.06	nd	nd
*Phenylpropene*	Methyl eugenol	52.93	1401	1402 C	0.3 ± 0.02 B	nd	0.5 ± 0.08 A	nd
*Sesquiterpene*	α-copaene	37.46	1378	1377 B	nd	nd	0.8 ± 0.08 B	1.9 ± 0.04 A
	α-gurjunene	39.40	1408	1409 C	nd	1.2 ± 0.19 A	nd	1.4 ± 0.08 A
	*Trans*-α-bergamotene	42.60	1438	1432 C	nd	2.4 ± 0.38	nd	nd
	α-guaiene	42.90	1440	1437 C	nd	0.4 ± 0.04 A	nd	0.4 ± 0.02 A
	β-caryophyllene	43.35	1408	1408 C	0.6 ± 0.01 B	1.2 ± 0.16 A	0.7 ± 0.39 AB	1.3 ± 0.03 A
	β-selinene	46.10	1496	1489 C	nd	2.2 ± 0.20 B	nd	3.2 ± 0.01 A
	γ-gurjunene	46.89	1479	1475 C	nd	3.2 ± 0.57 A	nd	3.3 ± 0.08 A
	α-muurolene	46.96	1499	1499 B	0.4 ± 0.16 B	nd	0.9 ± 0.01 A	0.1 ± 0.07 C
	δ-guaiene	47.30	n/a	-	0.3 ± 0.01 B	0.2 ± 0.02 C	6.6 ± 0.81 A	0.1 ± 0.00 D
	α-selinene	47.41	1505	1498 C	0.5 ± 0.04 A	nd	0.6 ± 0.05 A	nd
	β-bisabolene	47.52	1505	1500 C	1.1 ± 0.01 C	3.5 ± 0.42 A	1.8 ± 0.07 B	3.5 ± 0.06 A
	δ-cadinene	48.36	1500	1522 A	5.5 ± 0.46 C	20.4 ± 1.30 A	2.1 ± 0.20 D	16.1 ± 0.49 B
	Cis-Cadina-1(2),4-diene	49.00	1515	1524 C	nd	0.2 ± 0.01	nd	nd
	α-cadinene	49.21	1541	1537 C	nd	nd	nd	0.1
	Cycloisolongifolene	49.40	n/a	-	nd	0.5 ± 0.01 A	5.0 ± 0.02 A	nd
*Sesquiterpenoids*	α-Bergamotene	41.70	1438	1411 C	nd	0.4 ± 0.07 B	nd	1.2 ± 0.02 A
	Aromadendr-1-ene	43.52	n/a	-	0.8 ± 0.08 B	0.6 ± 0.06 C	2.0 ± 0.54 A	0.6 ± 0.03 C
*Sesquiterpenoids*	(+)- Aromadendrene	43.74	1454	1458 C	2.7 ± 0.00 A	nd	0.3 ± 0.05 B	nd
	Valencene	45.00	1715	1729 C	nd	nd	nd	0.2 ± 0.17
	Allo-aromadendrene	45.18	1635	1639 C	1.2 ± 0.02 C	1.9 ± 0.23 B	1.0 ± 0.04 D	3.0 ± 0.17 A
	α-humulene	45.99	1446	1455 B	nd	0.1 ± 0.01 A	nd	0.1 ± 0.01 A
	*Trans*-β-farnesene	46.36	1449	1456 C	0.4 ± 0.02 C	2.1 ± 0.25 A	1.4 ± 0.03 B	2.1 ± 0.02 A
	α-amorphene	46.50	1681	1693 C	nd	0.6 ± 0.07 A	nd	0.6 ± 0.01 A
	α-cubebene	46.55	1351	1352 C	nd	1.3 ± 0.85	nd	nd
	Viridiflorene	46.77	1570	1489 C	5.6 ± 0.08 A	3.5 ± 0.18 B	2.8 ± 0.07 C	3.1 ± 0.07 C
	β-gurjunene	48.43	1544	1430 C	0.3 ± 0.001 B	nd	4.8 ± 0.55 A	0.2 ± 0.19 B
	Cis-calamenene	50.08	1526	1510 C	0.4 ± 0.01 B	nd	0.7 ± 0.05 A	0.4 ± 0.01 B
	Geranyl acetone	50.38	1452	1453 C	0.2 ± 0.03	nd	nd	nd
	α-Calacorene	51.80	1538	1539 A	nd	nd	nd	1.5 ± 0.02
	Cadina-1(2),4-diene	54.04	1515	1524 C	nd	0.1 ± 0.07 B	0.4 ± 0.08 A	nd
	Viridiflorol	54.25	1593	1591 B	26.2 ± 1.24 A	17.9 ± 2.15 B	16.8 ± 0.31 B	17.0 ± 0.01 B
	Valeranone	55.00	1668	1666 A	nd	nd	nd	0.1 ± 0.26
	Epi-bicyclosesquiphellandrene	55.41	1488	1476 C	nd	nd	0.4 ± 0.06	nd
	T-Muurolol	55.57	1640	1644 C	0.3 ± 0.08 B	nd	0.8 ± 0.25 A	0.2 ± 0.00 B
	Pogostol	55.97	1656	-	nd	0.5 ± 0.05 B	2.9 ± 0.42 A	0.4 ± 0.07 B
	1,4-dimethyl-7-(1-methylethyl) azulene	56.30	1772	-	nd	0.2 ± 0.03 C	0.6 ± 0.17 A	0.3 ± 0.00 B
	Caryophyllenol II	57.11	1644	1655 C	nd	nd	0.2 ± 0.09	nd
*Terpene*	α-pinene	12.19	917	939 B	0.8 ± 0.001 B	0.9 ± 0.16 B	1.6 ± 0.25 A	1.9 ± 0.14 A
	Hexahydrofarnesylacetone	54.34	1844	1844 C	0.2 ± 0.08 B	nd	0.8 ± 0.03 A	nd
	Azulene	48.19	1311	-	0.3 ± 0.03 B	0.1 ± 0.02 C	1.9 ± 0.05 A	nd

Mean values (*n* = 3) along the rows with different letters are significantly different based on Duncan’s Multiple Range Test at *p* ≤ 0.05. All volatile compounds were tentatively identified by comparing the data of the MS with spectral data from the NIST v. 05 and the Wiley 275 mass spectral libraries. RT: Retention time (min), RI = Retention index, nd: implies not detected or below MS detection level, n/a: implies not available. * AH = Alkane hydrocarbons, Aroma. Hyds. = Aromatic hydrocarbons, BH = Bicyclic hydrocarbons, FAME = Fatty acid methyl esters, Exp = Experimental RI. ^†^ A = Adewinogo et al. [7], B = Najar et al. [8], C = Adams [15] and Babushok et al. [16], D = Qiu et al. [17].

### 2.5. Antifungal Activity

**Figure 1:**
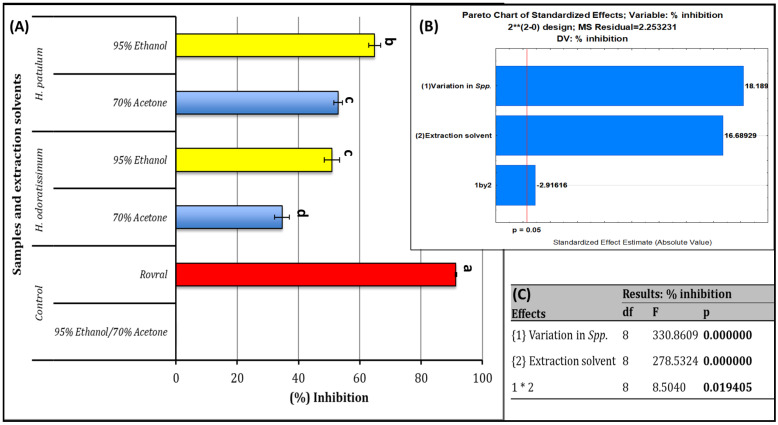
(**A**) Antifungal screening of aqueous acetone and ethanol extracts of *H. odoratissimum* (L.) and *H. patulum* by disk diffusion method against *Botrytis cinerea*. Mean values (*n* = 9) of microbial percentage inhibition with standard error bars. Different letters are significantly different based on Duncan’s Multiple Range Test at *p* ≤ 0.05. (**B**) Pareto analysis showing the effects of extraction solvent, variation in Spp., and interaction effects at 95% confidence indicated as the vertical red line. (**C**) Statistical parameters. * Colour only online.

The extracts obtained from *H. odoratissimum* and *H. patulum* using acetone and ethanol showed the highest percentage inhibition (*p* = 0.019, F = 8.50, DF = 8) against *B. cinerea* (Figure 1). The percentage inhibition of 65% and 51%, obtained by ethanol extracts of *H. patulum* and *H. odoratissimum*, respectively, were higher in comparison with the inhibition observed by acetone extracts (35% and 53%). The antifungal effects produced by the positive control Rovral^®^ WP (91%) against the *B. cinerea* was significantly higher than all extracts (*p* < 0.05) while no growth inhibition was observed for the negative controls (i.e., extraction solvents). These observations indicated that the extraction solvents alone did not play a role in the inhibitory effects of the crude extracts (Figure 1). The observed high antifungal effects from the ethanol and acetone extracts of *H. patulum* could be described by the abundant content of methyl cinnamate (cinnamic acid), as fatty acid methyl esters have been evidenced to poses high microbial effects [18]. In addition to that, high levels of δ-cadinene detected in acetone and ethanol extracts of *H. patulum* (20.4 ± 1.30% and 16.1 ± 0.49%, respectively) could also be implicated to these high antimicrobial effects observed. This compound has been included as a major compound of numerous plant extracts that showed high antimicrobial effects.

## 3. Discussion

The effects of extraction solvents and the type of *Helichrysum* species on the yield, chemical composition, and their activities were extensivity investigated using mainly the EO extracted from the plants [7,8]. The current study findings clearly showed that the SS was only influenced by the type of extraction solvent but not by the type of *Helichrysum species*. This could be allied with the nature of the extraction solvent and chemical composition of individual species. Several studies have reported the effect of solvents on the SS of plant materials. The results of this study agree with the reports by Matrose et al. [1] who showed that acetone as an extraction solvent resulted in higher SS compared to ethanol. The relatively higher SS yield obtained by aqueous acetone (70%) could be due to the polarity of the solvents, as highly polar solvents are known to result in higher extract yield compared to less polar solvents. Nawaz et al. [19] previously investigated the effect of solvent polarity on extraction yield and antioxidant properties of phytochemicals from bean (*Phaseolus vulgaris*) seeds. The authors also reported high extract yield obtained from polar solvents extracts and lower phenolic content compared to non-polar ones. The polarity of the solvents and the availability of the extractable plant constituents are the major contributing factors to variations in extraction yields. As the polarities of acetone and ethanol are 0.36 and 0.65, respectively [20], they were affected by the amount of water in the reaction matrix used in the current study. This further confirms that an extraction solvent with higher polarity is more effective for maximal extractable yield. According to Kim et al. [21], the addition of water to organic solvents increases the polarity of the solvents, hence the higher SS contents observed in the 70% acetone-based extracts than in 95% ethanol extracts used in the current study. Matrose et al. [1] further elucidated a significant correlation between high polar solvents and high extract yield. The authors reported a 40% reduction in the soluble solid yield of 95% ethanol than those of 70% acetone-based extracts. Therefore, based on the current study and the other studies stated above, aqueous acetone was the better extraction solvent to obtain a higher SS from *Helichrysum* species.

In this study, different extraction solvents showed a significant effect on the extraction yield of total polyphenol (TP) [22]. There are a few examples of literature about the TP content of *Helichrysum* species stem and leaf extracts. Matrose et al. [1] reported a 25% difference in the TP content of extracts obtained with 70% acetone and 95% ethanol of stems and leaves of *H. odoratissimum*. This discrepancy could be related to the polarity of the solvents used, as it was reported by Iloki-Assanga et al. [23] that the extractable phenolic compounds depend on the type of solvent used, its polarity index, and the solubility of phenolic compounds in the extraction solvent. Another study by Borges et al. [24] reported a reduction in active compounds obtained using 90% ethanol for pomegranate (peel, leaf, mesocarp, and seed) extracts compared to the 50% and 70% ethanol filtrates. The reduction in TP was associated with the highest solvent concentration of ethanol. Generally, high ethanol concentration leads to protein denaturation, preventing the dissolution of polyphenols leading to lowered extraction rate [25]. Ngo et al. [26] observed the highest extraction of TP using 50% (*v*/*v*) water–methanol, ethanol, and acetone for *Saptarangi* (*S. chinensis* L.). The authors revealed that 50% acetone extract had high levels of bioactive compounds (TPC 555 mg GAE g^−1^ CRE compared to that of 50% methanol and ethanol), thus confirming that 50% acetone was the most efficient solvent than ethanol. In addition, Iloki-Assanga et al. [23] reported the highest TPs from *Bucida buceras* extracted using pure acetone extraction solvent and the highest TPs from *Phoradendron califonicum* using water and pure methanol. The differences observed could be owed to the solubility and selectivity of plant compounds by solvents regardless of their similarity of the solvent’s polarity index.

The antioxidant capacity of *H. odoratissimum* extracts was significantly more affected than that of *H. patulum* by the extraction solvents. Results in the current study were supported by various studies who reported the influence of extraction solvents and discrepancy on the chemical profiles among plant species on the antioxidant capacity of different plants of the same genus. Even though the two species were obtained from the same agro-climatic zones, the differences observed in their antioxidant capacity could be influenced by a couple of factors (i.e., variability in the chemical composition of each plant species, selectivity of solvents, and the solubility of plant chemical compounds), which affects extraction yield [1,23,27]. A direct correlation was again confirmed in a study by Lim et al. [28]. The authors investigated the correlation between the extraction yield of *mangiferin* to the antioxidant activity, total phenolic, and total flavonoid content of *Phaleria macrocarpa* fruits, and a significant (*p* ≤ 0.05) correlation between the extraction solvent (methanol), method, extraction yield of mangiferin, and DPPH scavenging capacity was observed. In addition, Swarts [29] investigated the chemical profile of EOs obtained from *H. patulum*; the author reported on high antioxidant activity of the oils, which is allied to the presence of the phenolic constituent, arbutin. An earlier study by Takebayashi et al. [30] also reported weak antioxidant activity of arbutin. The authors, however, further discussed that the potency of the antioxidant of the compound to be directly dependent on the type of assay employed. Furthermore, there are several studies that showed the strong correlation between DPPH and TP content, which could explain the higher DPPH scavenging ability of *H. odoratissimum* than *H. patulum*. For instance, the results of this study indicated a significant (*p* ≤ 0.05) correlation between the total polyphenol content and the DPPH scavenging capacity of the extracts (Table 1).

The GC–MS analysis of this study identified higher concentrations of alkane hydrocarbons (tetradecane and hexadecane) in the *H. odoratissimum* extracts. These could be responsible for the higher antioxidant activity that was obtained in the *H. odoratissimum* extracts than *H. patulum* extracts. This could be supported by the findings of Ashraf et al. [31]. The authors compared chemical composition, antioxidant, and antimicrobial activities of EOs obtained from different parts (leaves and stems) of *Daphne mucronata* Royle. Their study reported a significantly high antioxidant activity in the EOs obtained from the leaves than that of the stems and linked this to alkane hydrocarbons. Pentadecane (12.75%), 2-methyl hexadecane (8.90%), 7,9-dimethyl hexadecane (8.90%), tetradecane (7.32%), 5-Propyl decane (6.16%), 2,3,5,8 tetramethyl hexadecane (5.81%), 2-methyl6-propyl dodecane (5.11%), and 5-methyl tetradecane (5.10%) were identified as the major constituents of the potent plant part. Generally, most of these compounds are reported to exhibit high antioxidant and high microbial effects [32]. In this study, the concertation of alkylbenzene and aromatic hydrocarbons were below the detected limit for ethanol extracts of both *H. patulum* and *H. odoratissimum*, suggesting the type of solvent could affect the volatile emission from the extracts. This could be due to the high concentration of ethanol (95%) in the extraction solvent; as stated by Feng et al. [25], high ethanol concentration leads to protein denaturation, preventing the dissolution of polyphenols leading to lowered extraction rate. In addition, acetone can dissolve both polar and nonpolar substances while ethanol amongst other solvents can only dissolve one or the other. The findings from this study are consistent with the results of De Canha et al. [33]. The authors reported a relatively high abundance of sesquiterpenoids and sesquiterpenes for the crude methanolic extracts of *H. odoratissimum* obtained from Kwazulu Natal. An earlier report by Gundidza and Zwaving [18] also reported similar results in a Zimbabwean *H. odoratissimum* with sesquiterpenoids (>22.6%) as major components. Similarly, Bougatsos et al. [34] reported the sesquiterpenoids (β-caryophyllene, 30.7% and 12.6%) as a major component of both *H. kraussii* and *H. rugulosum*, respectively. It is noteworthy that the phytochemistry reported for crude plant extract of *H. odorutissimum* and *H. patulum* in this study differs in the relative abundance of major and minor chemical classes from those reported in the literature for EOs. For instance, Najar et al. [35], identified α- and β-pinene (27.6% and 44.9%, respectively) as the major compounds in the EOs of *H. patulum*. It is, however, noteworthy that factors such as extraction solvent, plant parts, extraction method, sample type (crude extracts or EOs), geographical location, and species used play a crucial role on the extract yield and chemical constituents [1].

The antimicrobial inhibitory effects of *Helichrysum* spp. have been reported by previous studies [36]. The current study also confirmed marked growth inhibitory effects of both *H. odoratissimum and H. patulum.* Generally, *H. patulum* poses higher antifungal effects in comparison with *H. odoratissimum*. The findings of the current study were in support of the previous study by Louranse et al. [5]. The authors showed that *H. patulum* displayed antimicrobial activity against *Staphylococcus aureus* in the disc diffusion assay that was comparable to that of the positive control: ciprofloxacin. Phytochemical studies conducted in *H. patulum* extracts or Eos evidenced the presence of different compounds with high microbial activities. According to Najar et al. [35], sesquiterpenes are evident antimicrobials. The authors further noted that compounds such as the epoxide: caryophyllene oxide, sesquiterpenoid: *viridiflorol* for high antimicrobial activity thus could lead to the observation of the current study, as a high presence of the latter compounds were detected in all extracts tested. Khundu et at. [36] evaluated five cadinene derivatives (cadinan-3-ene-2,7-dione, 7-hydroxycadinan-3-ene-2-one, 5,6-dihydroxycadinan-3-ene-2,7-dione, cadinan-3,6-diene-2,7-dione, and 2-acetyl-cadinan-3,6-diene-7-one) fractionated from ethyl acetate extract of the leaves of *Eupatorium adenophorum*. The authors revealed that all compounds exhibit a high but selective antifungal activity. A previous study by Gonzalez et al. [37] identified δ-cadinene as a major compound in the methanol extracts of *Xenophyllum poposum*. The authors associated the high antifungal activity of the extracts with the presence of the later compound. As aforementioned, even though not in high concentration, β-selinene and *γ-gurjunene* were only identified in *H. patulum* extracts. High antimicrobial activity of these sesquiterpenes has been reported by Salleh et al. [38]. The authors further noted a direct correlation between the total polyphenol content of the extracts and antimicrobial activity.

## 4. Materials and Methods

### 4.1. Plant Material

**Figure 2:**
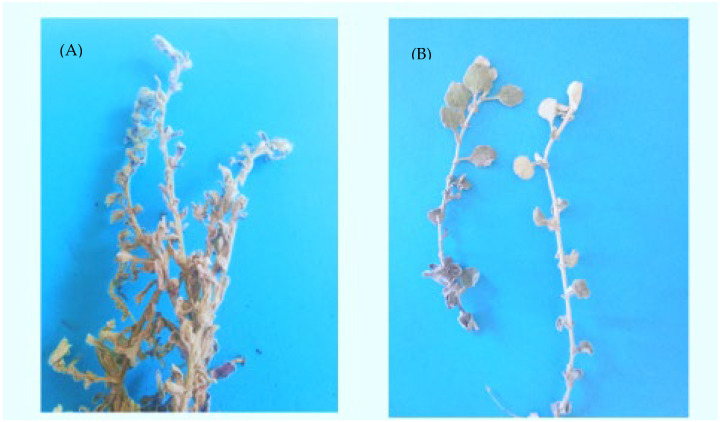
Pictures of the two *Helichrysum odoratissimum* (**A**), and *Helichrysum patulum* (**B**) were investigated in the study.

Fresh plant material (areal parts: leaves and stems) of *H. odoratissimum* L. [Sweet] (Voucher No: HO370) were collected from the Botrivier area in the Overberg region (Latitude: −34°13′0.02″ Longitude: 19°12′0″), Hermanus, South Africa and supplied by AfriNatural (Cape Town, South Africa). *H. patulum* was collected and identified (Voucher No: A9006) by the Tygerberg Nature Reserve on the Tygerberg hills in the northern suburbs (−33.8793° S: 18.5966° E) of Cape Town, South Africa. Figure 2 presents images of the *Helichrysum* spp. prior to processing. Plant material (leaves and stems) were immediately air-dried away from direct sunlight upon arrival and stored separately at room temperature in the dark at the Agri-Food Systems, Postharvest Pathology Laboratory at the ARC Infruitec-Nietvoorbij, Stellenbosch, South Africa until processing and extraction.

### 4.2. Processing and Extraction

Using a kitchen blender (91–357, Waring Blendor, Waring, Stamford, CT, USA), air-dried plant materials were roughly ground and further milled into finer powder. For each processed Helichrysum spp., 250 g was macerated in 1 L of 95% ethanol (Ethanolsa, Pretoria, South Africa) and 70% acetone (Ethanolsa, Pretoria, South Africa), as descried by Matrose et al. [1]. Macerated mixtures were placed at room temperature (24 °C) under vigorous shaking at regular intervals (8 h) for 72 h using a laboshake heavy load shaker (Gerhardt GmbH & Co. KG, Königswinter, Germany).

After maceration and vigorous shaking, the extracts were filtered through Whatman #4 (Whatman International, Ltd., Maidstone, UK) and transferred through a tea strainer into a clean Schott bottle. With the use of Sartorius^®^ Cellulose Nitrate (Cellulose Ester) Membrane Filters, 0.2 µm (0613114071301823, Sartorius Stedim Biotech, Göttingen, Germany) the filtrates were filtered further to eliminate microbial contamination. Filtrates were stored under aseptic condition at 4 °C until further analysis. All extractions were conducted in triplicate (*n* = 3).

### 4.3. Phytochemical Analyses

#### 4.3.1. Soluble Solids

A gravimetric approach was used to quantify the soluble solid (SS) content of the crude extracts by evaporating aliquots (20 mL) of each filtrate to dryness in the pre-weighed nickel moisture dishes on a steam bath. Thereafter, the moisturizing dishes were dried using a laboratory convection oven at 100 °C for 60 min. All measurements were carried out in triplicate (*n* = 3). Moisture dishes were cooled in a desiccator to room temperature then weighed and SS content calculated and expressed as (mg 100 mL^−1^).

#### 4.3.2. Total Polyphenol Analysis

BioTek Synergy HT multi-plate reader (BioTek Instruments, Winooski, VT, USA) was used to quantify the total polyphenol content of all the crude extracts. The Folin-Ciocalteu method by Singleton and Ross [39], which was further adapted for micro-palate reader format by Arthur et al. [40], was used in this study. Gallic acid (Sigma-Aldrich, Burlington, MA, USA) was used as the standard for the calibration curve ranging in concentration from 1 mg L^−1^ to 10 mg L^−1^. The samples stock solutions (1 mg mL^−1^) were diluted (300 µL sample diluted to a final volume of 1000 µL with deionized water) to obtain absorbance values within the range of the calibration curve. Gallic acid standards (20 µL) with the samples and the assay control (deionized water) were transferred in triplicate into a 96-well polystyrene flat-bottomed micro-plate (Greiner Bio-One, Frickenhausen, Germany). Folin-Ciocalteu’s reagent (10× diluted; 100 µL) and sodium carbonate (7.5% *w*/*v*; 80 µL, Merck, Darmstadt, Germany) solution were added into the reaction mixture, followed by mixing of the well contents using an Eppendorf Mix Mate micro-plate shaker (Merck, Darmstadt, Germany). The micro-plates were incubated for 2 h at 30 °C to allow for development of a blue-color complex resulting from oxidation of the phenolic compounds. The absorbance was measured at 765 nm and total polyphenol content (TP) in each extract was expressed as g Gallic Acid Equivalents (GAE) 100 g^−1^ dry weight of the leaves.

#### 4.3.3. Determination of Antioxidant Activity

The antioxidant activity of the plant extracts against 2,2–Diphenyl-2-picrylhydrazyl (DPPH) was determined using the method described by Arthur et al. [40]. The methanolic (Merck, Darmstadt, Germany) solution (0.05 mg mL^−1^) of DPPH (Sigma-Aldrich, Burlington, MA, USA) was prepared, covered with foil, and sonicated for 5 min. Using the BioTek Synergy HT multiplate reader (BioTek Instruments, Winooski, VT, USA), the DPPH-methanol solution absorbance value was adjusted to the range of 0.68–0.71 for assay standardization purposes. Trolox (Sigma-Aldrich, Burlington, MA, USA) was used as a calibration standard. A dilution range (1 µg mL^−1^ to 10 µg mL^−1^) of Trolox standard from stock solution (1 mM) was used to prepare a calibration curve in the reaction volume. Thirty microliters each of the Trolox standards, samples, and assay control (deionized water) were transferred in triplicate into corresponding wells of a 96 deep-well plate (Axygen Scientific Inc., Union City, CA, USA) and 270 µL DPPH solution (ca 0.7 absorbance value) was added.

To prevent evaporation of methanol (Merck, Darmstadt Germany), the sample-loaded plate was sealed with silicon mat. The contents in the 96 deep-well plate were then mixed for 30 s at 1650 rpm using an Eppendorf MixMate^®^ micro-plate shaker (Eppendorf AG, Hamburg, Germany). The micro-plates were incubated in a dark cupboard at room temperature for 2 h (to allow scavenging of DPPH by the antioxidants, leading to a decrease in absorbance of the free radical solution). Thereafter, 200 µL of the reaction mixture were transferred into corresponding wells of a polystyrene flat-bottom 96-well micro-plate (Greiner bio-one, Frickenhausen, Germany) and the absorbance measured at 515 nm using the BioTek Synergy HT multiplate reader The radical scavenging ability was expressed as µmol Trolox Equivalents (TE) g^−1^ extract.

### 4.4. Secondary Metabolites

Relative abundance of secondary metabolites of the *Helichrysum* spp. extracts were quantified by pipetting ≈5 mL of each extract into 20 mL solid-phase micro-extraction (SPME) vials. Each vial was allowed to equilibrate for 5 min at 50 °C in the CTC auto-sampler incubator at 250 rpm. Volatile compounds trapped in the headspace of the vails were extracted using the static headspace (SPME) method [41]. Subsequently, SPME fiber (50/30 µm) coated with divinylbenzene/-carboxen/-polydimethylsiloxane (DVB/CAR/PDMS) was exposed to the sample headspace for 10 min at 50 °C. After volatile extraction from SHS of the vials, desorption of the adsorbed compounds from the fiber coating was carried out in the injection port of the gas chromatography–mass spectrometry (GC–MS) for 10 min. The injector temperature was maintained at 250 °C.

Separation and quantification of the volatile compounds were performed on a gas chromatograph using Agilent 6890 N (Agilent, Palo Alto, CA, USA), coupled with an Agilent mass spectrometer detector Agilent 5975 MS (Agilent, Palo Alto, CA, USA). The system was equipped with a polar ZB-FFAP column (Model No.: ZB 7KM-G009-17), with a nominal length of 60 m, 0.25 μm internal diameter, and 0.5 μm film thickness. Helium gas was used as the carrier gas for these analyses at a flow of 2 mL min^−1^ with a nominal initial pressure of 196.0 kPa and an average velocity of 36 cm s^−1^. The oven temperature program was as follows: 35 °C for 5 min; and then ramped up to 50 °C at 3 °C min^−1^ and held for 3 min, and again ramped up to 120 °C at 3 °C min^−1^ with a 5 min holding time. Finally, the temperature was ramped up to 240 at 8 °C min^−1^ and held for 5 min. The MSD was operated in full scan mode and the ion source and quadrupole temperatures were maintained at 230 °C and 150 °C, respectively. The transfer line temperature was maintained at 250 °C. Compounds were tentatively identified by comparison of retention times (RT) and retention index (RI) with those registered in the National Institute of Standards and Technology (NIST v. 05, Gaithersbug, MD, USA) and the WHILEY 275 mass spectral libraries. Only compounds that occur across the triplicates and with correlation coefficient ≥ 80% from the NIST MS library were considered for further analysis. For quantification, the calculated relative abundances were used. This experiment was independently replicated in triplicate (*n* = 3).

### 4.5. Antimicrobial Activity Analysis

#### 4.5.1. Preparation of Test Fungal Pathogen

Fungal pathogen *B. Cinerea* isolated from infected plums was obtained from Agricultural Research Council (ARC)—Plant Protection Institute, Pretoria (Accession number: PPRI 7338). South Africa. *B. cinerea* was cultured on potato dextrose agar (PDA, Merck, Johannesburg, South Africa) at 25 °C for 3 days for mycelial plugs, and for 7 days to produce spores. The cultures of *B. cinerea*, were maintained on PDA slants at 4 °C. Conidia were harvested from the medium surface with sterile distilled water with Tween 80 (0.05% W/V) (Batch No.: 1006022, Merck, Darmstadt, Germany) and gentle agitating the plates to dislodge the spores. The final inoculum concentration was adjusted to 1 × 10^5^ conidia mL^−1^ using a haemocytometer (Marienfeld, Germany).

#### 4.5.2. Antifungal Activity

To determine the antifungal activity of the crude extracts against *B. cinerea*, a disc diffusion assay described by Matrose et al. was used [1]. Sterile Whatman #4 filter paper disks (4 mm, ø) were soaked in the perspective samples/standards/controls, i.e., (a) respective crude extracts concentration (250 mg mL^−1^) was selected for this investigation based on multiple preliminary study; (b) in 0.005 mg mL^−1^ of Rovral^®^ WP (Bayer Crop Science, Leverkusen, Germany) as negative control (standard); and (c) in the extraction solvents as positive controls. The PDA plates were spread-plated with 200 µL suspension of *B. cinerea* spores.

Inoculated plates were allowed to dry before the treated discs were aseptically placed at the centre. Each plate was sealed with parafilm (DEMIS flexible packaging, Demis, USA), and incubated at 25 °C for 5 days. The zone of inhibition on day 5 was measured using a digital calliper (MAC-AFRIC—Adendorff Machinery Mart, Johannesburg, South Africa), the percentage inhibition was calculated, and antifungal activity expressed as % Inhibition [1]. This experiment was independently replicated in triplicate and three times (*n* = 9).

### 4.6. Statistical Analysis

Data obtained were subjected to factorial analysis of variance (ANOVA) using Statistical software (vr. 13, StatSoft Inc. TIBCO Software Inc., Arlington, VA, USA). Effects of experimental factors: species (Spp.), extraction solvents (S), and their interactions (Spp. * S) on soluble solids (SS), total polyphenol (TP), antioxidant activity, and antifungal activities were analyzed. To test statistical differences between mean values, Duncan’s Multiple Range Test was at *p* ≤ 0.05. To describe the relationship between TP, antioxidant activity and SS, Pearson correlation matrix was used. Besides the antifungal activity, all experimental data obtained were in triplicate and the result presented as mean values (*n* = 3) ± standard deviation (SD).

## 5. Conclusions

This study showed that that extraction solvent plays a crucial role in the availability, bioactivity, and antifungal efficacy of bioactive compounds of *Helichrysum* Spp. Comparing between the antioxidant activities of *Helichrysum* Spp., *H. odoratissimum* had the highest free radical scavenging capacity for the extracts obtained with 70% acetone and 95% ethanol, while extracts from *H. patulum* showed lower scavenging activity. High antioxidant activity was consistent with the total polyphenol content. The GC–MS profiling of *Helichrysum odoratissimum* and *H. patulum* showed distinction variation in the secondary metabolites. Viridiflorol, a sesquiterpenoid with known antimicrobial activity, was most abundant in the 70% acetone crude extract of *H. odoratissimum*. Similarly, δ-cadinene was abundant in the 70% acetone crude extract of *H. patulum*. Fatty acid methyl ester (methyl cinnamate) was only detected in ethanolic extract of *H. patulum*. Thus, this study emphasises the importance of species variation in bioprospecting for new plant-based bioactive compounds.

## Figures and Tables

**Table 1:** Effects of variation in *Helichrysum* spp. and extraction solvents on the total polyphenol (TP), antioxidant capacity (TAC), and soluble solid (SS) yield on the crude extracts (on dry weight basis).

Sample	* Ext. Solvent	* TP (g GA 100 g^−1^)	* DPPH: TAC (µmol Trolox g^−1^)	SS (g 100 mL^−1^)
*H. odoratissimum*	Acetone	19.3 ± 0.76 ^A^	13,251.5 ± 700.55 ^A^	2.9 ± 0.003 ^A^
	Ethanol	14.5 ± 0.45 ^B^	9280.6 ± 704.01 ^B^	1.6 ± 0.002 ^B^
*H. patulum*	Acetone	4.9 ± 0.22 ^C^	5643.1 ± 259.09 ^C^	2.9 ± 0.001 ^A^
	Ethanol	4.4 ± 0.09 ^C^	2926.0 ± 138.48 ^D^	1.6 ± 0.001 ^B^
**Effects**		**TP (g GA 100 g^−1^)**		
		**DF**	**F Value**	***p* Value**
		8	2144.467	**0.000000**
Variation in *Spp*		8	102.389	**0.000008**
Variation in *Spp*.*Extraction solvent		8	62.886	**0.000047**
		**DPPH: TAC (umol Trolox/g)**		
		**DF**	**F value**	***p* Value**
Variation in *Spp*.		8	545.256	**0.000000**
Extraction solvent		8	125.091	**0.000004**
Variation in *Spp*.*Extraction solvent		8	4.397	0.069282
		**SS (g/100 mL)**		
		**DF**	**F Value**	***p* Value**
Variation in *Spp*.		8	1	0.404578
Extraction solvent		**8**	**1,226,185**	**0.000000**
Variation in *Spp*.*Extraction solvent		8	0	0.671623
***Pearson Correlation at p ≤ 0.05***				
**Variable**		**TP (g GA 100 g^−1^)**	**DPPH: TAC (µmol Trolox g^−1^)**	**SS (g 100 mL^−1^)**
TP (g GA/100 g)		1.00	0.97	0.21
DPPH: TAC (µmol Trolox g^−1^)		0.97	1.00	0.43
SS (g 100 mL^−1^)		0.21	0.43	1.00

Mean values (*n* = 3) along the rows with different upper-case letters are significantly different based on Duncan’s Multiple Range Test at *p* ≤ 0.05. *p* values and other parameter values in bold are statistically significant. * Ext. = Extraction, TP = total polyphenol content, DPPH: TAC = Total antioxidant capacity. Additional statistical parameters.

## Data Availability

The data that support the finding of this study are available from the corresponding author upon request.
